# Adapting the First Episode Psychosis Services Fidelity Scale in Asia: A Comprehensive Evaluation of Singapore's Early Psychosis Intervention Programme

**DOI:** 10.1111/eip.70131

**Published:** 2026-01-06

**Authors:** Amelia Sim, Quek Mable Jing Ting, Yi Lun Tay, Suying Ang, Donald Addington, Charmaine Tang, Swapna Verma

**Affiliations:** ^1^ Institute of Mental Health Singapore Singapore; ^2^ Department of Psychiatry University of Calgary, Cumming School of Medicine Calgary Canada

**Keywords:** early psychosis intervention, first episode psychosis services fidelity scale, service evaluation, Singapore mental healthcare

## Abstract

**Background:**

Fidelity assessments are crucial for ensuring evidence‐based delivery of first episode psychosis (FEP) services. The First Episode Psychosis Services Fidelity Scale (FEPS‐FS) is a validated tool for evaluating FEP programmes, but its application in Asian contexts remains unexplored. This study applies and adapts the FEPS‐FS to evaluate Singapore's Early Psychosis Intervention Programme (EPIP) in 2024, examining its contextual applicability within Singapore's healthcare framework.

**Methods:**

Fidelity was assessed at EPIP through programme data analysis, health record reviews, and interviews with staff, patients, and families. Three evaluators scored 37 items on a five‐point scale, with ratings determined by consensus. Two items were selectively added from an earlier FEPS‐FS version to address local contexts. Items rated ≤ 3 indicated areas needing attention. The FRAME methodology documented adaptations for local challenges, particularly in weight management and substance use interventions.

**Results:**

EPIP demonstrated high fidelity to evidence‐based FEP practices, particularly in programme structure and clinical processes. Twenty items achieved maximum ratings, including team integration, family engagement, and clozapine adherence. Lower ratings (≤ 3) identified gaps in participant ratios, psychiatrist caseload, and medication practices. Five items, primarily related to psychological and occupational therapies and substance use interventions, were unscored due to documentation differences, highlighting the need for tailored adaptations.

**Conclusion:**

This study demonstrates EPIP's strong adherence to evidence‐based practices while highlighting areas for improvement, particularly in resource allocation and documentation. The findings underscore the need for culturally sensitive adaptations of fidelity measures and innovative solutions to address challenges in early psychosis services within Asian healthcare systems. Future research should focus on developing structured documentation systems that balance standardisation with personalised care and explore strategies to enhance metabolic health interventions and substance use management in the local context.

## 1 Introduction

Psychotic disorders are a major cause of disability globally, with significant personal, familial, societal, and economic costs (Charlson et al. 2018). Early detection and timely provision of evidence‐based care during the initial stages of psychotic illness have been shown to improve clinical and functional outcomes (Correll et al. 2018). Consequently, specialised early intervention services for first‐episode psychosis (FEP) have proliferated worldwide in recent decades. Ensuring fidelity to established FEP service models is critical to delivering evidence‐based care effectively, achieving recovery‐oriented objectives, and mitigating the long‐term consequences of psychosis (Addington et al. 2018).

Fidelity in mental health refers to the extent to which a programme or service adheres to an evidence‐based model (Bond et al. 2000). Measuring fidelity enables the identification of service gaps, supports continuous quality improvement, and facilitates meaningful comparisons across programmes and contexts. Among the available fidelity scales, the First Episode Psychosis Services Fidelity Scale (FEP‐FS) (Addington et al. 2016) is widely recognised for its comprehensive structure and ease of implementation. Recent research mapping various fidelity scales to the FEPS‐FS has demonstrated its utility as a standardised tool within diverse early psychosis networks. This study found that the FEPS‐FS captured between 42% and 81% of components from other fidelity measures, underscoring its broad applicability and its ability to accommodate variations in programme delivery within learning health systems such as Early Psychosis Intervention Network (EPINET). The FEPS‐FS is explicitly designed for adaptation to local circumstances, with medication‐related items being country‐specific and population served criteria allowing for documented local incidence rates when available. Given its robust design, adaptability, such as its potential to be used as a dichotomous scoring system, and ability to provide consistent and actionable fidelity data, the FEPS‐FS serves as a useful instrument for ensuring high‐quality care and supporting quality improvement efforts in FEP services. It is for these reasons that we have applied the FEPS‐FS in this study.

The FEPS‐FS draws on 35 critical evidence‐based items of FEP services, assessing the services received by patients, the training received by the care providers, and how the team works together to engage and retain patients and deliver coordinated and evidence‐based care (Addington et al. 2018). Each item is rated on a five‐point behaviourally anchored scale ranging from 1 to 5. In the language of implementation, this ranges from 1, “Not implemented,” to 5, “Fully implemented.” In the language of quality, this ranges from 1, “Poor Quality,” to 5, “Excellent Quality.” In both languages, a 4 means good or satisfactory. It has been used to evaluate FEP services in several countries such as the United States, Canada, and Europe (Addington et al. 2018; Durbin et al. 2019) but to date, there is none done in Asia.

The contextual validity of fidelity scales developed in Western contexts should not be assumed when applying them in different healthcare systems (Kua and Rathi 2019). This is particularly relevant when examining the applicability of the FEPS‐FS in an Asian context, such as Singapore's EPIP. Understanding how FEP service fidelity may be influenced by contextual factors and health system characteristics is crucial for adapting early intervention models beyond Western settings. The concept of implementation adaptation supports this need for contextual adaptation (Wiltsey Stirman et al. 2019). Frameworks such as the Framework for Reporting Adaptations and Modifications‐Enhanced (FRAME) can be used to document adaptations, their rationale, and their relationship to fidelity. Applying such frameworks when using tools like the FEPS‐FS in different healthcare systems may help ensure their relevance and provide insights into necessary modifications for successful implementation of FEP services in varied contexts.

Singapore is a densely populated Southeast Asian city‐state with 6.11 million people (4.20 million residents, 1.91 million non‐residents). The resident population is predominantly Chinese (74.2%), Malay (13.7%), and Indian (8.9%), with other ethnicities comprising 3.2% (Department of Statistics Singapore 2024). The Singapore Mental Health Study 2016, a nationally representative, population‐based survey, reported a lifetime prevalence of schizophrenia and other psychotic disorders of 2.3% among community‐dwelling adults (Subramaniam et al. 2021).

Despite its advanced infrastructure and development, Singapore's mental healthcare system remains predominantly institution‐based (Kua and Rathi 2019). The Early Psychosis Intervention Programme (EPIP) was established in 2001 at the Institute of Mental Health (IMH), which is the sole tertiary psychiatric hospital in Singapore. EPIP is a comprehensive programme providing a range of pharmacological, psychosocial, and rehabilitation interventions for patients aged 16–40 years with FEP (Verma et al. 2012). The programme has reported positive outcomes in terms of remission rates, functioning, and service utilisation (Chong et al. 2005; Verma et al. 2012). It is important to note that EPIP's age range was expanded to 12–40 years shortly after this assessment was conducted. However, this study does not include data on patients aged 12–15, as the service extension to this younger age group was implemented after the initiation of our research.

This study built upon previous evaluations of EPIP, including a comprehensive programme evaluation conducted in 2012 (Verma et al. 2012). The earlier evaluation focused primarily on operational metrics such as the number of patients screened and accepted, duration of untreated psychosis (DUP), and broad outcome measures like symptomatic remission and functional recovery. These metrics provided valuable insights into the programme's reach and effectiveness at that time.

The current study advanced this evaluation approach by employing the standardised FEPS‐FS, offering a more nuanced assessment of EPIP's service delivery, focused on measures of structure and process while considering local contextual factors. Specifically, this study aimed to: (1) evaluate the level of fidelity of Singapore's only specialised FEP service using the FEPS‐FS, and (2) examine the relevance and applicability of the FEPS‐FS scale and its individual items in the Singaporean healthcare context. By doing so, we sought to not only assess EPIP's adherence to evidence‐based practices but also to contribute to the broader understanding of how fidelity scales developed in Western contexts could be applied and potentially adapted for use in Asian settings.

## 2 Methods

### 2.1 Setting

The study was conducted at the Singapore EPIP, which is based in the Institute of Mental Health (IMH), the only tertiary mental health institute in Singapore. EPIP accepts referrals of patients presenting with a first psychotic episode from both community and hospital sources. The programme spreads across both inpatient and outpatient settings. It provides 3 years of comprehensive case management and a range of evidence‐based pharmacological, psychological, and rehabilitation interventions. Interventions are delivered by a multidisciplinary team including psychiatrists, case managers, psychologists, social workers, occupational therapists, nurses, pharmacists, and peer support specialists.

### 2.2 Methodology

Health records review included a random sample of 10 active patients who had spent at least 1 year in the programme. The assessment team developed a randomization process for record selection, ensuring a representative sample across different stages of treatment. Interviewees were selected to represent diverse perspectives, including EPIP team leaders, psychiatrists, case managers, allied health staff, patients, and family members. Semi‐structured interviews lasting 45–60 min were conducted in private settings. Qualitative data from interviews were analysed thematically to complement quantitative findings. Inter‐rater reliability was ensured through independent ratings by three assessors, followed by consensus discussions to resolve discrepancies.

Using the FRAME methodology, we documented adaptations made to address local service delivery realities. We added item 36 (Individual and/or Group Interventions to Prevent Weight Gain) and retained item 37 (Motivational Enhancement or Cognitive Behavioural Therapy for Co‐Morbid Substance Use Disorder) as standalone items. These decisions reflected Singapore's healthcare structure where metabolic health and substance use management are delivered through specialised departments with distinct expertise. The FRAME approach supported documenting how care is organised in our context, where patients access different specialised services for comprehensive treatment, rather than assuming a fully integrated model. These adaptations allowed us to capture the actual service delivery pathways and identify opportunities for enhanced coordination between services.

### 2.3 Data Analysis

Fidelity to evidence‐based FEP practices at EPIP was assessed using an expanded version of the FEP‐FS. The scale comprises 37 programme‐specific items, each rated on a five‐point scale (Addington 2021). This included the original 35 items from the current FEPS‐FS, plus two additional items (items 36 and 37) from an earlier version of the scale, which were added to address specific local needs. Items rated 5 represented full implementation of evidence‐based practices. Items with ratings ≤ 3 were considered to represent areas for further attention.

The fidelity assessment was carried out from 1 June 2024 to 31 July 2024 and incorporated multiple data sources as shown in Table A1, Appendix A. Programme policy documents and administrative databases provided organisational and operational information. Health records review included a random sample of 10 active patients who had spent at least 1 year in the programme. The assessment team developed a randomization process for record selection. The assessment further included interviews with EPIP team leaders, psychiatrists, case managers, allied health staff, patients, and family members.

Inter‐rater reliability was ensured through a systematic process involving three raters: the principal investigator (AS), a member of the EPIP team (MQ), and an independent psychiatrist unaffiliated with the programme (YT). Ratings were initially performed independently, followed by a comparison to assess agreement levels. Discrepancies were discussed to reach a consensus or adjust the grading standards as necessary. The rating process adhered strictly to the scale manual and the accompanying fidelity review protocol (Addington 2021). Qualitative data from the health records and interviews were explored independently by all three raters to inform the ratings of the FEPS‐FS items where an item was not routinely documented in the health record. This served as an alternative data source to support the ratings as the appropriate data source.

## 3 Results

The compilation of fidelity ratings is presented in Table A2, Appendix A. To summarise, EPIP demonstrated high overall fidelity to evidence‐based practices for treatment of FEP. Twenty items achieved fidelity ratings of 5, particularly in areas of programme structure and clinical processes. Strong fidelity was evident in core team elements, including practicing team leader roles, psychiatrist involvement, and case manager assignments. The programme also showed high adherence in clinical processes such as comprehensive assessments and treatment planning, service integration through crisis intervention and inpatient communication, and family engagement through assessments, education, and support. The availability of clozapine for treatment‐resistant cases demonstrated alignment with evidence‐based practices.

Several other items received lower fidelity ratings (≤ 3), indicating areas for further study. These included item 2 (Participant/Provider Ratio), item 5 (Psychiatrist Caseload), item 10 (Age Range Served), and item 20 (Antipsychotic Dosing within Recommendations for Individuals with Psychosis). The programme's age range at the time of assessment (16–40 years, recently expanded after time of assessment to 12–40 years) differed from the FEPS‐FS target of 14–65 years. In terms of antipsychotic dosing, approximately 40% of patients received second‐generation antipsychotics (SGAs) at 50%–75% of institution‐approved guidelines.

The assessment process also revealed that five items could not be rated due to differences in how services were documented compared to the scale's requirements. These were item 24 (Cognitive Behavioural Therapy), item 28 (Supported Employment), item 29 (Supported Education), item 30 (Active Engagement and Retention) and item 37 (Motivational Enhancement or Cognitive Behavioural Therapy for Co‐Morbid Substance Use Disorder). The inability to rate these items reflected the complexity of understanding service delivery across EPIP's broader care network rather than the absence of these interventions. While the programme delivers these services through various pathways, including external specialised departments, the assessment process revealed challenges in fully capturing how these interventions are implemented across different organisational contexts. This highlighted opportunities for improved communication protocols to better track patient outcomes across the care system.

## 4 Discussion

The evaluation of EPIP using FEPS‐FS revealed strong performance in several key domains fundamental to effective early intervention services. EPIP demonstrated high fidelity in team structure and integration, patient and family engagement, and clinical processes. The programme's implementation of a unified model that seamlessly connects inpatient and outpatient services has contributed to consistent care delivery and strong team integration. Additionally, EPIP has achieved high rates of early engagement, with the majority of patients seen within 2 weeks of referral, reflecting effective community outreach and streamlined intake processes. This rapid patient engagement aligns with best practices for early intervention and contributes to the programme's overall effectiveness.

The current evaluation builds upon EPIP's 2012 programme evaluation by employing different assessment methods. While both evaluations assessed key outcomes such as DUP, symptomatic remission, and functional outcomes, the FEPS‐FS approach examines specific interventions and programme components in greater detail. This includes assessments of physical health monitoring practices, specific psychosocial interventions like CBT and supported employment, and the programme's approach to substance use treatment. The use of a standardised fidelity scale, in addition to operational metrics, allows for a more comprehensive evaluation of EPIP's service delivery. This approach may facilitate comparisons with other early intervention programmes globally, while still considering the unique aspects of Singapore's healthcare context.

### 4.1 Family‐Centered Care and Co‐Production

Research suggests that increased family involvement contributes to improved patient outcomes in Asian settings, aligning with cultural values of collective decision‐making and family‐centered care (Chakrabarti 2011). Early engagement of such support networks addresses a key predictor of treatment adherence and can mitigate disengagement risks, potentially leading to improved clinical outcomes (Doyle et al. 2014). EPIP has leveraged this factor, maintaining family participation rates of over 80% through flexible interventions that accommodate extended family networks and integrate family work across multiple team members. This approach not only capitalises on local cultural norms while adapting evidence‐based practices but also addresses the challenge of higher stigma associated with mental illness in many Asian countries, which can delay help‐seeking and impact engagement with services (Zhang, Si, et al. 2020a; Zhang, Sun, et al. 2020b).

Several EPIP case managers are trained in systemic therapy and receive regular supervision to enhance the use of these skills in patient care. This enables them to provide care that considers the patient's broader social context. Additionally, EPIP has a resource of a medical social worker who offers family therapy to patients identified by their case managers, ensuring targeted interventions that address both individual and family needs. Another key component of care is the Multiple Family Group Programme (MFGP), which has been shown to enhance insight, improve coping skills, and strengthen family relationships (Loh et al. 2023). These approaches capitalise on shared belief systems within Asian families such as common cultural practices and religious beliefs as coping strategies to aid recovery. A local study found that 68.9% of participants considered religion important in managing their illness, underscoring the value of integrating a family's religious perspectives into psychosocial interventions (Cetty et al. 2022). This culturally sensitive, family‐centered approach is effective in the Asian context, with MFGP participants reporting a sense of safety, mutual support, and improved understanding of psychosis (Loh et al. 2023).

Building on the family‐centered approach, EPIP has incorporated elements of co‐production into its service delivery model. This approach in mental healthcare involves collaboration between professionals and service users in designing and delivering services, aligning well with EPIP's emphasis on family involvement and cultural sensitivity. Projects and workshops involving co‐production and co‐facilitation have indicated modest improvements in mental well‐being, personal recovery, and social inclusion among participants. This integration of co‐production principles helps EPIP address potential power imbalances in mental healthcare and create services that better meet local community needs, potentially improving treatment engagement and outcomes (Lee et al. 2021).

### 4.2 Antipsychotic Prescribing Practices

All patients reviewed in the assessment were on SGAs, with approximately 40% receiving doses at 50%–75% of institution‐approved guidelines. While this may appear lower than expected, data from the Research on Asian Psychotropic Prescription Patterns for Antipsychotic (REAP‐AP) study have shown that among regional variations, Singapore's mean antipsychotic doses fall within the moderate range in East Asia (mean antipsychotic dose 420 ± 356 mg/day chlorpromazine equivalent) (Park et al. 2018). The REAP‐AP study further demonstrated that Asian populations show increased sensitivity to psychotropic medications (Dong et al. 2019; Xiang et al. 2016; Xiang et al. 2019). EPIP's approach to antipsychotic dosing reflects a careful balance between efficacy and tolerability, particularly crucial for their young patient population.

The importance of a balanced, low‐dose strategy is supported by results from a recent EPIP study on service disengagement which identified medication side effects as a primary reason for treatment discontinuation (Chua et al. 2024). Positive initial experiences with antipsychotic medication have been shown to significantly enhance long‐term service engagement and overall treatment outcomes (Lincoln et al. 2016). In addition, EPIP's high fidelity score in item 21: Clozapine for medication‐resistant symptoms suggests timely and appropriate use of evidence‐based prescribing for clozapine‐eligible patients (Tang et al. 2021). This rate compares favourably to the 41.7% delayed clozapine initiation rate observed in other local psychiatric settings, indicating EPIP's adherence to best practices in early psychosis intervention (Zheng, Chan, et al. 2022a; Zheng, Lee, et al. 2022b). This adherence to best practices is particularly noteworthy in the context of broader challenges in clozapine initiation. A local study found that in some settings, there can be significant delays in starting clozapine treatment, with a mean time of about 10 years and a median of 6 antipsychotic trials before clozapine is initiated (Law et al. 2023).

### 4.3 Metabolic Health Challenges and Interventions

The integration of specialised interventions for weight management remains a significant challenge as evidenced by the low fidelity score for item 36 (Individual and/or Group Interventions to Prevent Weight Gain). This is particularly concerning given the high prevalence of metabolic syndrome and cardiovascular risk factors among individuals with schizophrenia, contributing to elevated medical morbidity, mortality rates, and shortened life expectancy (Brown 1997; Hennekens et al. 2005; Saha et al. 2007). Research from various Asian countries highlights the substantial economic impact of metabolic complications in psychosis patients. In Hong Kong, individuals with psychosis who develop metabolic syndrome incur additional annual healthcare costs of approximately HKD 23000 (Chan et al. 2021). A Taiwanese study found that cardiometabolic complications increased annual healthcare costs by 32% in schizophrenia patients (Wu et al. 2020). While specific data for diabetic patients with psychosis in Singapore is limited, the average annual cost of diabetes management for a general Singaporean is estimated at SGD 2500, increasing substantially with complications (Ng et al. 2018). This impact is also particularly significant given the increased susceptibility of Asian populations to metabolic syndrome at lower BMI thresholds (Tan et al. 2019).

A previous study within EPIP revealed obesity as the most common comorbidity (23%) among patients, followed by hyperlipidemia (9%) and hypertension (6%) (Hui et al. 2019). These findings are further corroborated by another EPIP study which examined weight trajectories in first‐episode psychosis patients in Singapore. This research found that the majority of patients (72.6%) experienced significant weight gain over a 2‐year period, with most of the gain occurring in the first year of treatment (Chua et al. 2023). In response, EPIP had implemented a phased approach of workgroups between 2013 and 2019, focusing on four key areas: promoting physical health awareness, enhancing metabolic health monitoring processes, developing targeted interventions, and improving overall physical well‐being. In addition to existing outpatient group activities and caregiver engagement events, nutrition and fitness programmes were introduced for inpatients. Consequently, compliance improved in several areas of cardiometabolic monitoring, notably weight, waist circumference, and blood pressure measurements.

Despite these improvements, challenges persisted in certain areas. Blood test monitoring for fasting glucose and lipids remained inconsistent, and the reassessment of key risk factors for metabolic syndrome such as smoking habits, alcohol consumption, and family history of cardiovascular disease was irregular following the initial consultation (Hui et al. 2019; Tang et al. 2021). In addition, a significant number of in‐house physical health interventions were suspended due to national social distancing measures during the COVID‐19 pandemic. Since the lowering of the country's Disease Outbreak Response System Condition (DORSCON) level to Green in February 2023, these programmes have been gradually reinstated. This has been illustrated through Club EPIP, an outpatient facility providing psychosocial interventions, which implemented a phased approach to resuming services. The facility resumed with the reintroduction of its sports group in June 2023, followed by the life skills group in August 2023.

### 4.4 Addressing Substance Use and Metabolic Health

The fidelity assessment revealed significant gaps in addressing substance use among EPIP patients. Item 16 (Comprehensive clinical assessment at enrolment) showed that comorbid substance use was the most commonly uncompleted item during history‐taking. Furthermore, insufficient data limited the scoring of item 37 (Motivational Enhancement or Cognitive Behavioural Therapy for Co‐Morbid Substance Use Disorder). These findings highlight the need for more robust integration of substance use management within EPIP's service structure. This is particularly relevant given the increasing prevalence of substance use in Singapore, especially among young adults. A first‐ever survey on the prevalence of illicit drug use, conducted by IMH between April 2021 and July 2022, found that the starting mean age for consumption among Singaporeans and permanent residents was 15.9 years (Chua 2024). This early onset is reflected in recent trends, with the number of new drug abusers aged under 30 rising to 480 in 2023, a 20% increase from 2022 (Wong 2025). This trend is further exemplified by the rise in vaping among youth. Despite its illegal status, vaping offences increased by 58% from 2022 to 2023, with student‐related cases surging from fewer than 50 in 2018 to 800 in 2022 (Ong 2023; Tan 2024). Such statistics underscore the urgency of addressing substance use, particularly among young adults who are also at higher risk for FEP.

Moving forward, EPIP should adopt a systems‐thinking approach to address the complex interplay of metabolic health and substance use issues within its service structure. This perspective is rooted in health behaviour change theory which suggests that effective interventions must consider interconnected elements across individual, healthcare, and societal levels (Rutter 2002). For metabolic health, this approach could involve implementing standardised screening protocols, establishing clear pathways for intervention and follow‐up, and fostering closer collaboration with weight control clinics and in‐house dietitians. Based on EPIP's research on metformin for antipsychotic‐induced weight gain (Tang et al. 2021), both pharmacological interventions and lifestyle modifications should be considered, alongside adjusting medication protocols to favour weight‐neutral second‐generation antipsychotics as first‐line treatments.

National Addictions Management Service (NAMS) is a national service established in 2008 at IMH which provides comprehensive, multidisciplinary care for various addictions –(NAMS 2025). To address substance use challenges, closer integration between the departments of EPIP and NAMS is necessary. This could involve structured communication protocols and shared care pathways tailored to FEP patients with comorbid substance use issues. By aligning collaborative efforts with evidence‐based clinical standards, EPIP can create a more integrated and effective care model addressing both metabolic health and substance use. This approach could enable seamless referrals, coordinated treatment plans, and shared expertise across different domains, allowing for more comprehensive initial assessments and consistent follow‐ups that address the gaps identified in the fidelity assessment.

### 4.5 Addressing Resource Constraints

The low fidelity scores on item 2 (Participant/Provider ratio) and item 5 (Psychiatrist Caseload) reflect broader challenges within Singapore's healthcare landscape. Mental health services in Singapore are predominantly tertiary based, with IMH serving as the country's sole tertiary psychiatric hospital (Ministry of Health 2023). This centralised structure, while efficient in some respects, creates unique pressures on service delivery. According to the World Health Organization (WHO) Mental Health Atlas, Singapore's mental health workforce density falls below the average for high‐income countries, with just 4.6 psychiatrists and 9.7 psychologists per 100 000 population (WHO 2023). These structural constraints impact service delivery, organization, and documentation across the mental health sector, including specialised programmes like EPIP. Mental health services across Asia face comparable challenges in managing caseloads and maintaining optimal clinician‐to‐patient ratios. For instance, Hong Kong's EASY programme, which shares many structural similarities with EPIP, has reported ongoing struggles with balancing service demand and workforce capacity (Tang et al. 2010). Similarly, a study from India highlighted significant regional disparities in mental health service provision, with many areas falling well below recommended clinician‐to‐patient ratios due to resource constraints and workforce shortages (Gautham et al. 2020). These patterns underscore a common dilemma: how to balance limited resources with the need for intensive, individualised care in certain patient populations.

To address these challenges, EPIP could explore several innovative strategies. Collaborating with primary care providers and polyclinics to create a shared care model could enhance early detection and reduce the burden on tertiary services. Leveraging digital health technologies for symptom monitoring and low‐intensity interventions could allow clinicians to focus on more complex cases and critical periods of care. The HOPE‐S (Health Outcomes via Positive Engagement in Schizophrenia) study at IMH has already demonstrated promising results in this area. Using digital phenotyping and machine learning algorithms, the study achieved high sensitivity (91.4%) and specificity (95.3%) in predicting adverse events such as hospital readmissions, emergency room visits, and clinical deterioration in schizophrenia patients (Creighton et al. 2024; Rashid et al. 2021). Such technology could potentially enable early intervention and more targeted care, reducing clinician workload. Additionally, establishing partnerships with community organisations and peer support networks could provide supplementary psychosocial support, further alleviating clinical burden.

### 4.6 Navigating Programme Evaluation: EPIP's Strengths and Opportunities

The fidelity assessment identified several items that could not be scored due to insufficient data as required by the FEP‐FS framework. This limitation, however, should be contextualised within EPIP's existing comprehensive monitoring system. Since its inception, EPIP has maintained rigorous tracking of operational indicators through the Ministry of Health's semi‐annual reporting framework, as mandated by the National Mental Health Blueprint (NMHBP). These indicators encompass service accessibility, engagement rates, clinical outcomes, and satisfaction metrics. For instance, EPIP has consistently demonstrated high performance across these measures, with over 90% of patients seen within 2 weeks of referral, three‐year follow‐up rates exceeding 90%, and significant clinical improvements.

To address these measurement challenges while maintaining personalised care, EPIP could develop a more structured documentation system that captures the nuances of tailored interventions while improving their measurability. The FRAME methodology could guide this process, highlighting opportunities for adaptations to enhance the scale's relevance in our context. For instance, items related to Supported Employment and Supported Education could be modified to better reflect EPIP's flexible, individualised approach to psychosocial interventions. This approach could help balance the need for standardised evaluation with EPIP's commitment to personalised care, ensuring that fidelity assessments accurately capture the essence of EPIP's service delivery model.

## 5 Conclusion

This fidelity assessment of EPIP using the FEP‐FS highlights both the strengths and areas for improvement in delivering evidence‐based early psychosis care within Singapore's unique healthcare context. The study demonstrates EPIP's high fidelity to many core elements of early intervention services, particularly in team structure, patient engagement, and clinical processes. However, it also identifies key challenges, especially in resource allocation, documentation practices, and the management of metabolic health and substance use issues.

The findings underscore the importance of balancing fidelity to established models with necessary cultural adaptations. The use of the FRAME methodology to document adaptations proved valuable in understanding how the FEPS‐FS could be tailored to better reflect the realities of service delivery in an Asian context. This approach not only facilitated a more nuanced evaluation of EPIP but also contributed to the broader understanding of how fidelity scales developed in Western contexts can be effectively applied and adapted for use in diverse healthcare systems.

As early psychosis intervention continues to evolve in Asian settings, ongoing evaluation and quality improvement efforts are crucial. Future research should focus on:

1. Developing culturally sensitive fidelity measures that can accurately capture the nuances of early psychosis care in diverse contexts
2. Exploring innovative solutions to address resource constraints, particularly in managing caseloads and enhancing specialised interventions.
3. Implementing and evaluating structured documentation systems that balance standardisation with the need for personalised care
4. Strengthening the integration of metabolic health and substance use interventions within early psychosis services.
5. Investigating the long‐term impact of culturally adapted early intervention models on patient outcomes.

In conclusion, this study not only provides valuable insights into the current state of early psychosis intervention in Singapore but also offers a roadmap for future improvements. By continuing to refine and adapt fidelity measures and service delivery models, early intervention programmes like EPIP can better meet the needs of people experiencing psychosis, ultimately improving long‐term outcomes and quality of life for this population.

## Funding

The authors have nothing to report.

## Conflicts of Interest

The authors declare no conflicts of interest.

## Data Availability

The data that support the findings of this study are available from the corresponding author upon reasonable request.

## Appendix A Tables A1 and A2

**TABLE A1:** Data sources.

Data type	Description	Number of sources
Programme and administrative data	Programme policy	1
	Administrative data	1
Health records review	Patients in EPIP between 1 and 2 years	3
	Patients in EPIP 2–3 years	4
	Patients in EPIP > 3 years	3
Staff interviews	Team leader	1
	Psychiatrists	2
	Case manager lead	1
	Case managers	2
	Occupational therapist	1
	Psychologist	1
Patient and family interviews	Patients	2
	Families	2
Staff meeting observations	Team meetings	2

**TABLE A2:** Ratings per item.

Item number	Item name	Rating	Key data sources	Comments, if any
1	Practicing Team Leader	5	– Interview with team leader	
2	Participant/Provider Ratio	3	– Administrative data, interviews with team leader and case managers	
3	Services Delivered by Team	4	– Interviews with team leader, psychiatrists and case managers – Programme policy	– Programme's exclusion criteria includes substance‐related psychosis including drugs and alcohol. – Comorbid substance use management is carried out by a separate department (NAMS) upon referral.
4	Assigned Case Manager	5	– Interviews with team leader and case managers – Interviews with patients and family members – Administrative data – Programme policy	– Every patient is assigned a case manager upon entry into the programme.
5	Psychiatrist Caseload	3	– Administrative data – Interviews with psychiatrists	– The time interval of repeat appointments with a psychiatrist range from weeks‐months dependent on patient's needs. – Range of psychiatrists' caseload is 40–46 for 0.2 FTE.
6	Psychiatrist Role on Team	5	– Interviews with psychiatrists	
7	Weekly Multi‐Disciplinary Team Meetings	5	– Health record review – Interviews with team leader, psychiatrists and case managers – Programme policy	
8	Explicit Admission Criteria	5	– Administrative data – Programme policy – Interviews with team leader, psychiatrists and case managers	– Inclusion criteria: Patients with first episode psychosis aged between 12 and 40 years old – Exclusion criteria: Psychosis due to a general medical condition, substance‐related psychosis including drugs and alcohol, intellectual disability, current remand/forensic cases
9	Population Served	5	– Interviews with team leader – Administrative data – Census data	– 95% of incident cases within age range of 15–45 are admitted to FEP service.
10	Age Range Served	3	– Programme policy	– Program served an age range of 19–40 at time of assessment. – Age range has since lengthened to 12–40 at time of writing.
11	Duration of FEP Program	4	– Programme policy	– Patients who need a longer period of care have the service extended on a case‐to‐case basis beyond 3 years.
12	Targeted Health/Social Service/Community Groups	5	– Administrative data – Interviews with team leader and case managers	– Training of Ministry of Education (MOE) counsellors once a year – Training for social workers on Psychosis with National University of Singapore – Outreach to schools and community partners via CHAT, a youth mental health service – Incidental outreach to community social workers and counsellors via co‐creation workshops
13	Early Intervention	4	– Administrative data	– 38.9% of patients have received inpatient care prior to FEPS admission.
14	Timely contact with referred individual	4	– Administrative data	– 76.9% of patients were offered an in‐person appointment within 2 weeks of service receiving referral.
15	Family Involvement in Assessments	5	– Health record review – Interviews with team leader, psychiatrists and case managers – Interviews with family members and patients	
16	Comprehensive clinical assessment at enrolment	4	– Health record review – Interviews with team leader, psychiatrists and case managers – Interviews with family members and patients	– 80% of clinical assessments had 6–8 items completed, with co‐morbid substance use being most commonly uncompleted.
17	Psychosocial needs assessed for care plan	5	– Health record review – Interviews with team leader, psychiatrists and case managers – Interviews with family members and patients	– Primary care access is universal in our local healthcare setting.
18	Clinical Treatment Plan/Care Plan after Initial Assessment	5	– Health record review – Interviews with team leader, psychiatrists and case managers – Interviews with family members and patients	– In the event of treatment refusal leading to a significant risk of harm to self or others, formalised inpatient treatment may be administered under the Mental Health and Treatment Act.
19	Antipsychotic Medication Prescription	5	– Health record review – Interviews with team leader, psychiatrists and case managers – Interviews with family members and patients	– Patients' preferences are brought up during discussions with the clinician before commencement of antipsychotic medication. – Subsequent clinician reviews assess for suitability, efficacy and side effects of medications
20	Antipsychotic Dosing within Recommendations for Individuals with Psychosis	3	– Health record review	– 40% of patients had doses at 50%–75% of institution‐approved guidelines for SGAs. – All patients reviewed were on SGAs. – Dosages tended to be lower for tolerability in a younger and Asian population.
21	Clozapine for Medication‐Resistant Symptoms	5	– Tang et al. (2016)	– 4.3% of patients (no distinction between mood disorder or Schizophrenia) were on Clozapine during the service period of 3 years.
22	Patient Psychoeducation	5	– Health record review – Interviews with team leader, psychiatrists and case managers – Interviews with patients	– Patients are offered group and/or individual psychoeducation. – Individual psychoeducation is customised to a patient's profile and can be delivered by various members of the treating team, including the treating psychiatrist, case manager, peer support specialists, psychologist, pharmacist and occupational therapists. – Printed materials for psychoeducation are available and written in recovery language. – Training and supervision are given to case managers on how to provide psychoeducation in a process orientated manner. – A structured 6 session co‐production workshop is conducted on a regular basis.
23	Family Education and Support	5	– Health record review – Interviews with case managers – Interviews with family members	– Families are offered group and/or individual psychoeducation. – Printed materials for psychoeducation are available in three languages. – Multi‐family groups are held annually. – Several case managers are trained to take on a systemic lens and supervised to include a systemic focus in their management. – One case manager is trained in Behavioural Family Therapy. Materials for this have been adapted to the programme instead of a manualized manner.
24	Cognitive Behavioural Therapy (CBT)	Not rated	– Interviews with team leader, case managers and psychologist – Interviews with patients	– Insufficient data on the % of patients receiving CBT or of the number of sessions given. – Patients are referred for psychological therapy on a needs basis. – In addition, the form of therapy may not be specifically CBT. The type of therapy is decided upon each patient's individual requirements. These may include Family therapy, Solution focused therapy, Acceptance and Commitment Therapy, among others.
25	Supporting Health	5	– Health record review – Interviews with team leader, psychiatrists and case managers – Interviews with patients and family members	– Primary care access is universal in Singapore's healthcare setting.
26	Annual Comprehensive Assessment	5	– Health record review – Interviews with team leader, psychiatrists and case managers – Interviews with patients and family members	– Substance use item was not documented in 80% of assessments.
27	Services for Patients with Substance Use Disorders (SUD)	4	– Health record review – Interviews with team leader, psychiatrists and case managers – Interviews with patients' family members	– Patients with SUD are referred to a separate department (NAMS) for co‐management
28	Supported Employment (SE)	Not rated	– Interview with occupational therapist – Interviews with team leader and case managers – Interviews with patients and family members	– Occupational therapists perform the SE role. – Some patients are referred to an occupational therapist within the service, while others are referred to the external institution's occupational therapy department (OTD). Management may defer depending on which setting patient is receiving SE intervention from. – No available information on tracking of supervision, caseloads and number of employer contacts.
29	Supported Education (SEd)	Not rated	– Interview with occupational therapist – Interviews with team leader and case managers – Interviews with patients and family members	– Refer to item 28 comments.
30	Active Engagement and Retention	Not rated	– Interviews with team leader and case managers – Interviews with patients and family members	– Numbers of community/home visits are not tracked. – At least 1 home visit is made per patient during their time with the programme. Some patients have more on a needs basis.
31	Patient Retention	5	– Programme administrative data – Interview with team leader	– Inactive cases continue to be retained in the programme.
32	Crisis Intervention Services	5	– Programme policy – Interviews with team leader and case managers	– Patients are provided with 24 h crisis outreach services per day, 7 days per week, through separate Emergency Services within the institution.
33	Communication between FEP and Inpatient Services	5	– Administrative data – Programme policy	– The team encompasses both inpatient and outpatient care. The assigned case manager remains the same regardless of area of care, hence communication and discharge planning during and after hospitalisation are seamless.
34	Timely Contact After Discharge from Hospital	5	– Administrative data – Programme policy	– Refer to item 33 comments
35	Assuring Fidelity	3	– Administrative data	– Programme has standards but does not use a fidelity scale. Programme monitors ≥ 4 quality indicators linked to standards.
36	Individual and/or Group Interventions to Prevent Weight Gain	2	– Interview with occupational therapist – Interviews with team leader and case managers – Interviews with patients	– Weight is taken at each outpatient clinic visit and feedback provided via the case manager and/or the psychiatrist. Referrals to dieticians are made on a needs basis.
37	Motivational Enhancement (ME) or Cognitive Behavioural Therapy (CBT) for Co‐Morbid Substance Use Disorder (SUD)	Not rated	– Interviews with team leader, case managers	– Patients with SUD are referred to a separate department (NAMS) for co‐management. – Lack of access to information as to how many patients with SUD receive at least 3 sessions of ME or CBT.

*Note:* The following table provides the score for 37 fidelity items from the First‐Episode Psychosis Services Fidelity Scale (FEPS‐FS) used in the assessment of EPIP. Each item has received a score between 1 (indicating a low level of fidelity) to 5 (indicating a high level of fidelity), as well as the data sources from which the score was determined and any relevant contextual information to explain the score.
